# Prognostic Impact of Neutrophil-to-Lymphocyte Ratio in Ischemic Stroke

**DOI:** 10.3390/jpm14121149

**Published:** 2024-12-10

**Authors:** Santhiago Calvelo Graça, Tainá Mosca, Vivian Dias Baptista Gagliardi, Wilma Carvalho Neves Forte, Rubens José Gagliardi

**Affiliations:** Santa Casa de São Paulo School of Medical Sciences, São Paulo 01224-001, Brazil; santhiagocalvelo@hotmail.com (S.C.G.);

**Keywords:** ischemic stroke, inflammation, prognosis, neutrophil

## Abstract

**Background/objective**: Studies suggest that the neutrophil/lymphocyte ratio (NLR) may be a prognostic marker for different diseases with inflammatory components. This study aimed to quantify the NLR in individuals affected by different subtypes and severities of ischemic stroke and associated it with risk factors and treatment, and compared the results with data from healthy individuals. **Methods**: Clinical and laboratory data from medical records of patients over 18 years of age, victims of ischemic stroke, were collected. Data included leukocyte count and subtype, topography, risk factors, treatment and severity of stroke. For comparison, the number of leukocytes in healthy individuals was also quantified. NLR was determined by dividing the number of neutrophils by the number of lymphocytes. **Results**: A total of 218 patients were included, 194 stroke patients and 24 healthy individuals. Among all stroke patients, 45% had NLR values > 4 and 35% had values between 2 and 4; otherwise, 71% of healthy individuals had NRL < 2. The data also showed that the greater the severity of the stroke, measured by the NIHSS scale, the higher the NLR, at 24 and 72 h after the stroke. Among the stroke subtypes evaluated, the one with the lowest NLR values was small vessel stroke. Finally, the risk factors for stroke, its topography and treatment were not associated with NLR values. **Conclusions**: NLR is associated with stroke severity but does not correlate with stroke risk factors, topography, and treatment. The NLR may serve as a marker of stroke severity.

## 1. Introduction

Stroke is a common disease that affects approximately 15 million people worldwide each year. Among individuals affected by a stroke, one-third succumb to mortality, and another third are left with permanent neurological disabilities [1]. In Brazil, it is the second leading cause of death and the primary cause of disabilities [2,3].

Studies suggest that inflammation plays a crucial role in stroke, being related to the development and rupture of atherosclerotic plaques and the worsening of cerebral ischemia [4,5,6]. The inflammatory process in the central nervous system (CNS) that occurs in stroke involves different types of cells, such as microglia, T lymphocytes, and neutrophils. This process is a consequence of tissue damage caused by ischemia and the release of substances such as damage-associated molecular patterns (DAMPs) and pathogen-associated molecular patterns (PAMPs) [6,7,8]. Microglia activated by neuroinflammation derivatives can phagocytize lytic cells and pathogens. However, an excess of this function can be harmful to the CNS during a stroke. Additionally, cells present in the ischemic core of the damaged tissue, such as glial cells, may undergo lysis, ceasing to control the inflammation generated by tissue damage. Peripheral blood cells attempt to fulfill this function, resulting in the proliferation and accumulation of peripheral microglia and leukocytes [7].

Neutrophils represent the first type of cells that infiltrate CNS, crossing the blood–brain barrier (BBB) within a few hours after the injury and reaching their peak concentration in the first three days after ischemia. Regardless of how neutrophils reach the injured area, the presence of these cells has effects on the prognosis of the disease [6,9].

Upon reaching the ischemic area, neutrophils are activated and release various substances, such as neutrophil elastase, which increases vascular permeability and hemorrhage, in addition to positively regulating the diapedesis of these leukocytes [10]. Neutrophil extracellular traps (NETs), a defense mechanism against antigens formed by the extravasation of DNA segments, histones, and proteolytic enzymes, participate, along with platelets, in a cycle of activation present in ischemic strokes [11]. Thus, this mechanism increases ischemic foci in the CNS, worsening the condition of ischemic stroke. For all these reasons, the presence of neutrophils in the CNS has been associated with a worse prognosis for stroke.

In addition to neutrophils, the role of lymphocytes has also been studied in stroke. Lymphocytes, cells of the adaptive immune system, produce various cellular mediators that modulate the inflammatory response [12]. Cytokines synthesized by lymphocytes can act as either protectors or inducers of the progression of ischemic injury in stroke [8,13]. Among these cytokines, IL-6 induces the progression of the injury, while IL-10 has a protective role in the progression of the injury [14]. Low lymphocyte levels in the CNS or peripheral blood can be predictive of little neurological improvement during the first week and a poor functional outcome after three months. The suggested mechanism is that lymphopenia reflects an increase in baseline levels of cortisol and sympathetic tone, which increases the production of pro-inflammatory cytokines, worsening ischemia [15].

Recently, it has been suggested that the neutrophil-to-lymphocyte ratio (NLR) is a good marker for systemic inflammation. NLR is determined by the division between the absolute number of neutrophils and lymphocytes in peripheral blood, and can be determined by a complete blood count [5]. The accuracy of NLR in predicting severity is well-documented for cardiac events, cancers, sepsis, multiple sclerosis, infectious diseases, and other inflammatory conditions [16]. Pikija et al. suggest that in-hospital mortality and the evolution of ischemic stroke in 90 days could be related to NLR: a high value at hospital admission could be a predictor of intracerebral hemorrhage and three-month mortality; high NLR values in acute ischemic stroke seem to suggest an unfavorable outcome due to neutrophil activation and lymphocyte suppression by stress [17]. Thus, NLR may be associated with neurological deterioration and possibly an unfavorable prognosis for stroke.

The aim of this study is to quantify the NLR in individuals affected by ischemic stroke and compare the results with data from healthy individuals of the same age group. Additionally, the study looks at whether there is a correlation between NLR values and risk factors associated with ischemic stroke and its progression.

## 2. Materials and Methods

### 2.1. Patients and Control Subjects

The present study encompasses both retrospective and prospective characteristics.

In the prospective stage, blood samples from the healthy individuals were collected and absolute values of neutrophils and lymphocytes were determined for quantification of NLR.

In the retrospective stage, data were collected from the medical records of patients who were victims of ischemic stroke and subsequently followed up in a specialized outpatient clinic. The data obtained refer to type and severity of stroke, associated risk factors of stroke, the treatment and the total peripheral blood leukocyte count.

The individuals who participated in the study were over 18 years old, of both sexes, and were patients of the Central Hospital and Hemotherapy and Hematology Unit of a tertiary hospital located in the downtown area of São Paulo (Brazil). All individuals participating in the study had their data collected between January 2017 and December 2023. Written informed consent was obtained from all study participants. The study was conducted according to the guidelines of the Declaration of Helsinki, and approved by the Ethics and Research Committee of Santa Casa de Misericórdia de São Paulo (CAAE 53823921.0.0000.5479; 02/22/2022).

The study participants were divided into 2 groups: stroke victims and healthy individuals.

The stroke group was formed by individuals suffering from ischemic stroke, treated in emergency care up to 24 h after the ischemic event. Exclusion criteria for this group included patients with other types of strokes, intracerebral hemorrhages prior to the study, those who experienced head trauma at the time of stroke, or had seizures; and concomitant myocardial infarction, metabolic decompensation, or any other relevant factor that could alter the course of evolution, irrespective of the stroke. Patients with autoimmune diseases, ongoing neoplasms, who were undergoing immunosuppressive therapy, and those with signs and symptoms of infection were also excluded. Patients whose time of onset of symptoms could not be reliably determined were also excluded.

The healthy individuals, blood donors of the Hemotherapy and Hematology Unit, were used as the control group for the study. These individuals were previously selected among the blood donors, whose blood bags were effectively approved. This group consisted of individuals with no history of stroke or transient ischemic attack, smoking, alcoholism, obesity, infectious or inflammatory disease, ongoing neoplasms or any other chronic comorbidity such as renal or hepatic insufficiency, hypertension, diabetes, and dyslipidemia, among others. In order to evaluate them, questionnaires were carried out prior to blood collection, regarding the aforementioned comorbidities, as well as blood tests to check for any changes, analyzed by a certified outsourced laboratory of the hospital.

### 2.2. Clinical Evaluation

The diagnosis of ischemic stroke was based on clinical features. Computed tomography (CT) scans of the brain were performed within 24 h of admission to exclude patients with primary intracerebral hemorrhage and other conditions that mimic stroke. To assess the degree of atherosclerosis, bedside carotid ultrasound was performed in patients and control subjects with conditions that allowed the examination. In addition, electrocardiogram was performed to evaluate cardiac abnormalities that could be related to the cause of the stroke.

After evaluating the aforementioned exams, strokes were divided into subgroups based on the TOAST scale (Trial of ORG 10172 in Acute Stroke Treatment). The TOAST scale is the most commonly used ischemic-stroke subtype classification system worldwide and has five stroke subtype categories: atherosclerotic, cardioembolic, small vessel, cryptogenic, and stroke of other determined etiology [18]. This study included the subtypes atherosclerotic, cardioembolic, small vessel, and cryptogenic.

Regarding the severity of neurological impairment, the National Institutes of Health Stroke Scale (NIHSS) was used for assessment. The NIHSS scale is based on the person’s performance, which is assessed through 11 categories, such as sensory and motor capacity [19]. Throughout the care, qualified health professionals applied the scale at the time of admission and every 24 h to assess whether there was improvement or worsening of the condition. To stratify into different subgroups of the results obtained, subgroups were formed with scores ≤ 5, 6 to 10, 11 to 15, and >15, since this division has already been used in other studies [20,21] and no work has proposed to compare it with NLR values.

The topography of the stroke was also a characteristic evaluated. The CNS can be divided based on the “tentorium cerebelli”, forming two intracranial compartments, one supratentorial and one infratentorial [22]. Based on this division, we also evaluated whether this division was associated with the data obtained.

Individuals with ischemic stroke were also divided according to the presence or absence of thromboembolic treatment. Patients with up to 4 and a half hours of ischemia, after excluding intracranial bleeding, underwent thrombolysis with alteplase at a dose of 0.9 mg/kg, with a maximum of 90 mg [2,23]. During the procedure, patients were constantly evaluated, to detect any intracranial bleeding early.

### 2.3. Determination of Neutrophil Lymphocyte Ratio (NLR)

The NLR was derived from the total number of neutrophils/mm³ divided by the total number of lymphocytes/mm³ from peripheral blood.

The determination of the total number of each leukocyte in healthy individuals, was carried out by multiplying the percentages of each type of leukocyte found in the blood smear, multiplied by the total number of leukocytes found with Tuerk’s Solution (Newprov, Pinhais, PR, Brazil), in a counting chamber.

Blood smears were made using fresh peripheral blood, collected in tubes containing heparin (BD, Franklin Lakes, NJ, USA). A small drop of blood (9 µL) was placed on the pre-cleaned, labeled slide and smears were made along the slide. The smear staining was carried out with fast panoptic stain kit (Instant Prov, Newprov, Pinhais, PR, Brazil). The percentage of each leukocyte was determined by a count of a fixed number of 200 cells on the smear.

The total number of blood leukocytes was determined in a chamber slide count of preparation of blood plus Tuerk’s solution (1:20). In this preparation, the erythrocytes are hemolyzed by Tuerk’s solution and the leukocyte nuclei are stained by the dye.

The determination of the NLR, in individuals affected by stroke, was obtained from hemogram data recorded in the enrolled patients’ medical records. NLR was determined by dividing the total number of neutrophils by the number of lymphocytes.

### 2.4. Statistics

Statistical analyses were performed by the statistics service of the institution where the work was carried out, using the SPSS Statistics 24.0 software (SPSS Inc., Chicago, IL, USA). Data normality was assessed using the Shapiro–Wilk test. Since the data did not present a normal distribution, the data were represented by the median and range (minimum–maximum) and the values were compared using the nonparametric Mann–Whitney test. A significance level of 5% was adopted in all tests.

Categorical data were expressed as percentage, and compared using the Chi-Squared test.

## 3. Results

A total of 952 individuals were initially selected, and 734 were excluded after applying the exclusion criteria. Among the patients affected by ischemic stroke, 103 were male and 91 were female, with a mean age of 65 years ± 15. Among the healthy blood donors, the mean age was 38 years ± 9, with 9 females and 15 males. Of the total number of patients, 66 (34%) were victims of cardioembolic stroke, 54 (28%) of atherothrombotic stroke, 26 (13%) of small vessel stroke and 48 (25%) of cryptogenic stroke. Among the patients who suffered from ischemic stroke, 161 (83%) had a supratentorial stroke and 33 (17%) had an infratentorial stroke. The risk factors associated with stroke, prevalent in the individuals studied, were, in order from most observed to least observed, arterial hypertension, diabetes mellitus, smoking, previous stroke, alcoholism and dyslipidemia.

**Figure 1:**
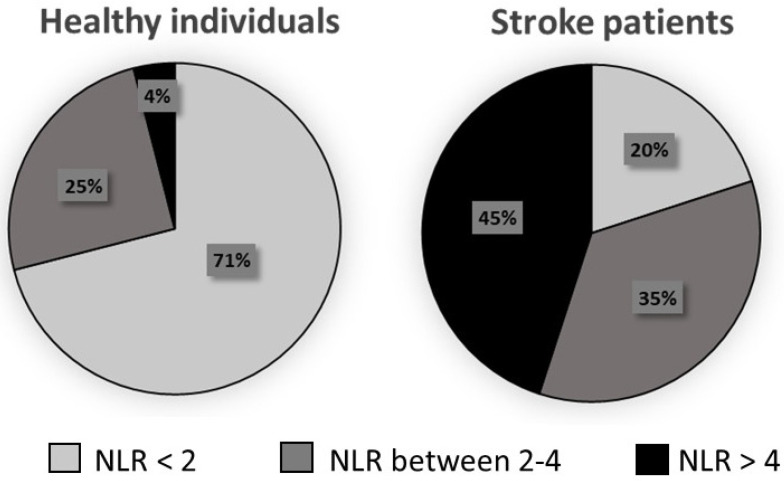
Graphic showing the percentages of healthy individuals (blood bank donors; *n* = 24) and stroke patients (stroke victims; *n* = 194) in the neutrophil-to-lymphocyte ratio (NLR) ranges (<2, between 2 and 4, and >4). The percentages found in each NLR range (<2, 2–4, 4), in healthy individuals and those affected by stroke, were compared using the Chi-Squared test. All values were different.

In Figure 1, the percentage of healthy individuals and stroke patients within NLR ranges (≤2, between 2 and 4, and ≥4) is depicted. NLR values were discriminated as ≤ 2 (physiological values [24]), between 2 and 4 and ≥4 (risk of death [5]). It was observed that among the 194 stroke patients, 80% had NLR values exceeding 2, while only 29% of healthy individuals possessed the same NLR value.

**Figure 2:**
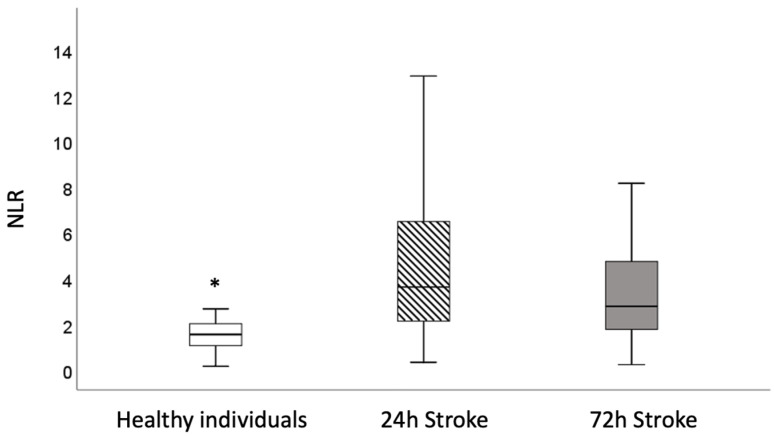
Box plot of neutrophil-to-lymphocyte Ratio (NLR) in healthy individuals (blood bank donors; *n* = 24) and patients 24 and 72 h after onset of ischemic stroke (stroke victims; *n* = 194). Values expressed as median and minimum and maximum values. Outliers not shown. * *p* < 0.001. Mann–Whitney test preceded by Shapiro–Wilk normality test.

In Figure 2, it is possible to see the NLR values of healthy individuals compared with the values found in stroke victims. It is possible to observe that, regardless of the time after stroke (24 or 72 h), healthy individuals present lower NLR values when compared to stroke patients.

In Table 1, the NLR values are shown for cardioembolic, atherothrombotic, small vessel, and cryptogenic types of stroke. It is observed that small vessel strokes had a lower NLR among the subtypes of stroke. No statistically significant difference was observed in NLR values among patients with different risk factors, thrombolysis and stroke topography.

**Figure 3:**
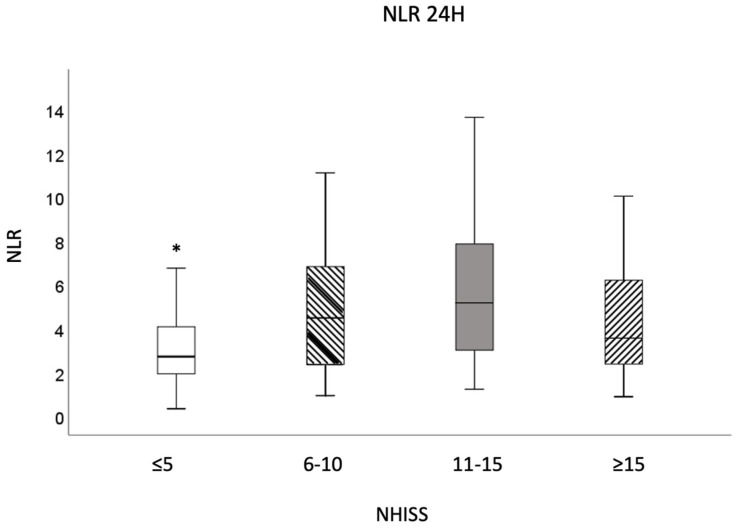
Box plot of neutrophil-to-lymphocyte ratio (NLR) according to the severity of stroke (NIHSS scale) at 24 h after medical care. Values expressed as median and minimum and maximum values. Outliers not shown. * *p* ≤ 0.05, in the comparison between stroke severities (NIHSS). Mann–Whitney test preceded by Shapiro–Wilk normality test.

In Figure 3 it is possible to observe that the severity of the stroke at 24 h, according to the NIHSS scale, was associated with the NLR: less severe strokes (NIHSS ≤ 5) had NLR values that were statistically lower than those observed in patients with more severe strokes (NIHSS > 6).

**Figure 4:**
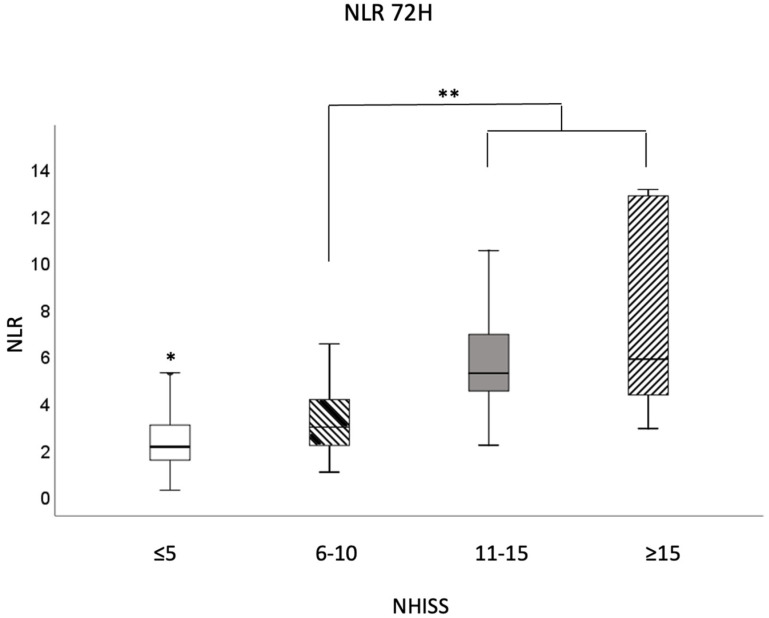
Box plot of neutrophil-to-lymphocyte ratio (NLR) according to the severity of stroke (NIHSS scale) at 72 h after medical care. Values expressed as median and minimum and maximum values. Outliers not shown. *; ** *p* ≤ 0.05, in the comparison between stroke severities (NIHSS). Mann–Whitney test preceded by Shapiro–Wilk normality test.

In Figure 4, it is possible to note, similarly to what was noted in Figure 3, that stroke severity is associated with high NLR values. It was observed that at 72 h after stroke, patients with stroke severity ≤ 5 presented values statistically lower than those found in other severities. In addition, strokes classified as 6–10 also presented NLR values statistically lower than those found in severities 11–15 and >15.

## 4. Discussion

The present paper is one of the few studies that analyses the NLR for the types of stroke, their severities and, risk factors and compares them with healthy individuals. It was observed that patients with ischemic stroke have higher values of NLR compared to healthy individuals. Additionally, these NLR values were associated with certain subtypes of ischemic stroke. Among these, the small vessel subtype, also known as lacunar stroke, was linked to lower NLR values in the first 24 and 72 h, compared to the values of other subtypes. The severity of the ischemic condition was also correlated with NLR values, as patients with less severe strokes exhibited lower NLR values.

In a relatively similar way to that observed in the present study, it is well-described in the literature that NLR is a good marker for inflammatory processes, considering NLR values above 2 as indicators of systemic inflammation and values below 2 as physiological [5,24,25,26].

Furthermore, some studies have correlated NLR with a worse outcome of ischemic stroke, either due to increased local inflammation, a higher association with pneumonia, or delirium after stroke [27]. Regarding severity, Xue 2022 demonstrated a relationship between NLR values and the severity of ischemic stroke, dividing patients into a moderate condition (NIHSS < 7) or severe condition (NIHSS ≥ 7). In this study, elevated NLR values are associated with a worse neurological condition in the first six hours [28]. Ying 2021 shows something similar, but the moderate condition was considered with an NIHSS ≤ 8, and severe >8 [29]. Differently, the present study evaluated NLR in four different NIHSS categories (≤5, 6 to 10, 11 to 15, and >15 [20,21]), finding significant differences between these categories, expanding the analysis of NLR on the severity of stroke and suggesting that NLR could be a prognostic marker for stroke severity.

Bearing in mind that the maximum concentration of neutrophils occurs between the first and third days after the onset of ischemic stroke [2], therefore, the assessment of the NLR should be carried out during this period. The results of this study demonstrated that lower NLR values are associated with less severe ischemic strokes, with NIHSS ≤ 5 in the first 24 h, in line with the literature results [28]. In the first 72 h, NLR also proved to be efficient in inferring the severity of the disease, as there was a difference in NLR values in the NIHSS ≤ 5, between the 6 and 10 and the >11 categories.

It was also observed that, among the subtypes of stroke, according to the TOAST scale, the subtype with lower NLR values is the small vessel stroke, which correlates with lower systemic inflammation. In the literature, no articles have been found, until now, studying the possible correlation between the etiology of stroke and NLR. It is known that ischemic conditions, leading to neuronal death, release various markers that activate the immune system, allowing the passage of neutrophils into the central nervous system (CNS) by breaking the blood–brain barrier [2,6]. Therefore, the smaller the ischemic area, the smaller the area of neuronal death, and consequently, the lower the inflammation. Among the subtypes of ischemic stroke, small vessel strokes involve a smaller ischemic area [30].

Regarding the affected region (supratentorial or infratentorial), the findings did not show a significant difference. No articles studying the effects of stroke topography on NLR values were found in the literature, to date. So, the present study is a pioneer in showing that there is no correlation between NLR and stroke topography.

Ischemic conditions are closely related to pre-existing comorbidities, which act continuously until, at a certain point, they cause the cessation of blood flow [2,31]. However, this study did not show correlation between NLR values and stroke comorbidities, such as hypertension, diabetes mellitus, dyslipidemia, or previous stroke. The influence of these risk factors on the magnitude of neuroinflammation did not differ, as NLR values were similar. Comorbidities possibly lead to inflammation through endothelial dysfunction and free-radical production, promoting cell damage and the release of DAMPs, which, in turn, feed back into the inflammatory process by activating more cells in this system. Consequently, there is a weakening of the blood–brain barrier, an occurrence of cell death, and a higher chance of thromboembolic events, leading to stroke [32]. Thus, neuroinflammation is closely associated with stroke and influenced by pre-existing comorbidities, which act chronically. However, the influence of each comorbidity, apparently, is not related to the magnitude of inflammation, as measured by NLR.

Similarly to the mentioned risk factors, habits and vices also influence vascular conditions, such as stroke [2]. However, this study did not show an association between NLR and habits and vices. Chronic smoking, for example, by producing free radicals, increases the inflammatory process and is associated with atherosclerosis [33], considered a risk factor for stroke. Chronic alcoholism has a toxic effect on glial cells, myelin, and microvasculature, increasing neuroinflammation [34], but these factors did not modify NLR.

The generally recommended treatment for ischemic stroke involves the use of thrombolytics such as recombinant tissue-type plasminogen activators, with alteplase and tenecteplase being the most common [35,36]. In this study, there was no difference in NLR values between patients who underwent thrombolysis and those who did not, either in the first 24 or 72 h. Previous studies demonstrated, unlike what was observed in this study, that on the first day after thrombolysis, there is an increase in the number of neutrophils and a reduction in the number of lymphocytes, which could justify an increase in NLR [37]. The release of matrix metalloproteinases (MMP-9) by neutrophils, reaching the site of ischemia, both pre- and post-thrombolysis, worsens local injury and may attract more of these cells to the ischemic region. Therefore, thrombolysis or conservative treatment may increase NLR, a fact not observed in this study [31].

This study has some limitations, such as the age difference between the mean age of healthy individuals and those affected by stroke, with a younger age group of blood donors forming the healthy group. Lastly, another limitation is the smaller sample size of patients with small-vessel and cryptogenic stroke, compared to those with cardioembolic and atherothrombotic stroke. With a larger number of patients, the observed difference in NLR in small vessel stroke may be even greater than found.

Nevertheless, this study is one of the few that evaluates NLR in both stroke subtypes and related risk factors, across all severity ranges (NIHSS), as well as the topography and treatment, and compares this with healthy individuals.

## 5. Conclusions

Patients affected by ischemic stroke exhibit an NLR with higher values than those observed in healthy individuals. Patients with more severe strokes have higher NLR compared to patients with less severe conditions. The observed NLR was lower in the case of small vessel strokes among patients with cardioembolic, atherothrombotic, small-vessel, and cryptogenic strokes.

This study suggests that peripheral blood NLR in stroke patients may serve as a prognostic marker for the condition and potentially assist in therapeutic decisions.

## Figures and Tables

**Table 1:** Neutrophil-to-lymphocyte ratio (NLR) according to the subtype, topography, risk factors and treatment of stroke.

		24 h	72 h
**Subtype of stroke**	Cardioembolic (*n* = 66)	3.82 (3.99–5.46)	2.96 (3.47–5.80)
	Atherothrombotic (*n* = 54)	3.69 (4.17–6.45)	3.04 (3.16–5.84)
	Small vases (*n* = 26)	2.53 * (2.47–4.16)	1.97 * (1.75–2.65)
	Cryptogenic (*n* = 48)	4.10 (3.87–6.59)	2.57 (2.19–4.27)
**Stroke topography**	Supratentorial (*n* = 161)	3.9 (0.4–18.6)	2.9 (0.3–31.0)
	Infratentorial (*n* = 33)	3.0 (1.3–16.3)	2.2 (0.9–11.3)
**Risk factors**	Systemic Arterial hypertension (*n* = 128)	3.6 (0.4–18.1)	2.8 (0.3–31.0)
	Diabetes mellitus (n = 63)	3.6 (1.3–18.1)	2.9 (0.9–31.0)
	Smoking (*n* = 36)	3.6 (0.4–10.1)	2.6 (0.3–13.1)
	Previous stroke (*n* = 31)	3.8 (1.3–10.6)	2.9 (0.9–7.0)
	Alcoholism (n = 29)	3.6 (1.3–10.1)	2.8 (0.9–6.1)
	Dyslipidemia (n = 22)	4.9 (1.3–16.4)	4.3 (1.1–12.8)
**Treatment**	Thrombolyzed (n = 73)	3.9 (1.3–18.6)	2.9 (0.9–31.0)
	Not thrombolyzed (*n* = 116)	3.7 (0.4–16.3)	2.6 (0.3–16.2)

Median and minimum–maximum values of neutrophil-to-lymphocyte Ratio (NLR) according to the subtype, topography, risk factors and treatment of stroke. * *p* ≤ 0.05, in the comparison at the same time. Mann–Whitney test preceded by Shapiro–Wilk normality test.

## Data Availability

The data presented in this study are available on request from the corresponding author (the data are not publicly available due to privacy or ethical restrictions).
